# The influence of gender on the epidemiology of and outcome from sepsis associated acute kidney injury in ICU: a retrospective propensity-matched cohort study

**DOI:** 10.1186/s40001-024-01651-8

**Published:** 2024-01-17

**Authors:** Wei Jiang, Lin Song, Yaosheng Zhang, Jingjing Ba, Jing Yuan, Xianghui Li, Ting Liao, Chuanqing Zhang, Jun Shao, Jiangquan Yu, Ruiqiang Zheng

**Affiliations:** 1 Medcial College, Yang Zhou University, Yangzhou, 225001 China; 2 Department of Critical Care Medicine, Clinical Medicine College, Yangzhou University & Intensive Care Unit, Northern Jiangsu People’s Hospital, Yangzhou, 225001 China; 3 Department of Echocardiography, Northern Jiangsu People’s Hospital, Yangzhou, 225001 China; 4 School of Clinical and Basic Medicine, Shandong First Medical University & Shandong Academy of Medical Sciences, Jinan, 250000 China; 5 Department of Cardiology, the Second Affiliated Hospital, Shandong First Medical University & Shandong Academy of Medical Sciences, Taian, 271000 China

**Keywords:** Sepsis, Acute kidney injury, Sepsis associated acute kidney injury

## Abstract

**Purposes:**

The influence of gender on the epidemiology of and outcome from SA-AKI in ICU has not been fully clarified. Our aim is to elucidate these differences.

**Methods:**

This study included adult patients with sepsis in MIMIC IV (V 2.2), and propensity matching analysis, cox regression and logistic regression were used to analyze gender differences in incidence, mortality and organ support rate.

**Results:**

Of the 24,467 patients included in the cohort, 18,128 were retained after propensity score matching. In the matched cohort, the incidence of SA-AKI in males is higher than that in females (58.6% vs. 56.2%; *P* = 0.001).males were associated with a higher risk of SA-AKI (OR:1.07(1.01–1.14), *P* = 0.026;adjusted OR:1.07(1.01–1.14), *P* < 0.033).In SA-AKI patients, males were associated with a lower risk of ICU mortality(HR:0.803(0.721–0.893), *P* < 0.001;adjusted HR:0.836(0.746–0.937), *P* = 0.002) and in-hospital mortality(HR: 0.820(0.748–0.899), *P* < 0.001;adjusted HR:0.853(0.775–0.938), *P* = 0.003).there were no statistically significant differences between male and female patients in 1-year all-cause mortality (36.9% vs. 35.8%, *P* = 0.12), kidney replacement therapy rate (7.8% vs.7.4%, *P* = 0.547), mechanical ventilation rate 64.8% vs.63.9%, *P* = 0.369), and usage of vasoactive drugs (55.4% vs. 54.6%, *P* = 0.418).

**Conclusions:**

Gender may affect the incidence and outcomes of SA-AKI, further research is needed to fully understand the impact of gender on SA-AKI patients.

## Introduction

Sepsis is a condition caused by the immune response dysfunction of the host due to infection, leading to multiple organ dysfunction [1]. There are over 18 million cases of sepsis worldwide each year, and it is a dangerous condition with high mortality rates [2]. The kidneys are vulnerable organs in sepsis, and statistics show that 60% of sepsis patients will develop Acute Kidney Injury (AKI), with a 50% mortality rate among AKI patients. Even for survivors, there is an increased risk of developing chronic kidney disease, often requiring long-term dialysis treatment [3]. Sepsis-associated acute kidney injury (SA-AKI) is independently associated with poor prognosis for patients, imposing significant burdens on both individuals and society [4]. Currently, apart from kidney transplantation, there are no effective treatment options available. This is due to the complex and yet unclear pathophysiological mechanisms underlying SA-AKI. SA-AKI presents as a complex clinical syndrome that can result in various clinical phenotypes and subtypes based on interactions between genotypes and exposures. It is precisely this heterogeneity that complicates the treatment and assessment of therapeutic efficacy for SA-AKI [5]. Identifying specific genotypic profiles and clinical phenotypes in patients has important implications for precision medicine treatments as well as evaluating treatment effectiveness.

Gender, as a genetic modifier in biology and disease [6, 7], leads to differences in the prevalence, prognosis, and treatment of many diseases between males and females [8–12]. However, the role of gender in SA-AKI is still unclear. On one hand, excessive inflammatory response and immune suppression during sepsis are closely related to SA-AKI [13]. Sex steroids have binary effects on immunity; estrogen may have a protective effect in an infectious environment while testosterone may be harmful due to its immunosuppressive properties [14]. This may reduce the susceptibility of females to SA-AKI. On the other hand, endothelial dysfunction activation during sepsis is closely associated with SA-AKI. Compared to males, females exhibit significant activation of endothelial cell function during sepsis [15], which may increase their susceptibility to SA-AKI. In conclusion, the differences in incidence rate, organ support rate, and prognosis of SA-AKI based on gender have not been fully elucidated and further research is needed.

Our research primarily aims to analyze SA-AKI patients in international large-scale databases. Firstly, we aim to evaluate the impact of gender on the incidence rate of SA-AKI in critically ill sepsis patients. Secondly, we aim to assess the influence of gender on organ support rates among SA-AKI patients. Lastly, we aim to evaluate the effect of gender on both short-term and long-term survival rates for these patients.

## Method

### Data source

This is a retrospective cohort study using the MIMIC-IV (version 2.2) database to analyze different populations. The MIMIC-IV database is a publicly available multi-parameter intensive care database provided by the Massachusetts Institute of Technology (MIT) [16]. It includes critically ill patients admitted to the ICU at Beth Israel Deaconess Medical Center in Boston, Massachusetts, from 2008 to 2019. Since this study is based on analysis of a third-party anonymous public database and has obtained institutional review board approval in advance, ethical review is not required. To access this database, we have completed the online training course and Protecting Human Research Participants exam offered by the National Institutes of Health (No. 54780440).

### Study population

This study selected adult septic patients who were admitted to the ICU from the MIMIC-IV database from 2008 to 2019. Patients with severe chronic kidney disease (CKD), defined as CKD stage ≥ 4 or estimated glomerular filtration rate (eGFR) < 30 mL/min/1.73m^2^, and patients undergoing long-term dialysis treatment were excluded.

### Identification of sepsis

According to the definition of SEPSIS-3, we identified patients with confirmed or suspected infection and a Sequential Organ Failure Assessment (SOFA) score increase of two or more [1]. We determined the clinician's recognition of suspected infection through two simultaneous events in the electronic health records: (1) prescription of antibiotics, and (2) ordering specific fluid cultures. These two events need to occur within a specific time frame and have the following options: In option 1, fluid culture is performed first, and antibiotic use must be initiated within 72 h. In option 2, antibiotic dosing is administered first, and fluid culture must be completed within 24 h. We excluded all antibiotics given as a single dose in the operating room. We also excluded antibiotics that were not accompanied by fluid cultures. We included fluid cultures from multiple sites: abdomen, bronchoalveolar lavage, blood, bone marrow, cerebrospinal fluid, catheter/device tips, pleural effusion, skin/tissue samples, stool, and urinary tract. Cultures types included bacterial, fungal, viral. We assumed a SOFA score of zero prior to ICU admission. If individual components of SOFA were missing, no contribution was made to the total score [17]. The daily total SOFA scores were calculated, and an increase of two points within 24 h was considered abnormal [18]. Considering difficulties in interpreting neurological SOFA when sedation therapy is being concurrently administered, it was not included in the overall scoring category [19].

### Identification of acute kidney injury

The diagnostic criteria for AKI follow the standards of Kidney Disease: Improving Global Outcomes (KDIGO): an increase in serum creatinine (Scr) exceeding 26.5 μmol/L (0.3 mg/dl) within 48 h; an increase in serum creatinine by more than 50% from baseline, lasting for 7 days; urine output less than 0.5 ml/(kg·h), lasting for more than 6 h [20]. The minimum Scr value available within the first 7 days prior to admission is used as the baseline Scr [21, 22]. When pre-admission Scr is not available, the first measured Scr upon admission is used as the baseline Scr [23]. Either urine-based or creatinine-based criteria, or a combination of both, are used to determine if a patient meets KDIGO AKI criteria.

### Identification of sepsis-associated acute kidney injury

After determining sepsis and acute kidney injury separately, we applied the definition of SA-AKI from the ADQI 28 working group. We compared the diagnosis day of sepsis with the diagnosis day of AKI. If AKI occurs within 1–7 days after the diagnosis of sepsis, patients are classified as SA-AKI according to ADQI criteria [5]. If AKI occurs before sepsis, patients do not meet the definition of SA-AKI.

### Outcomes

The main outcome was the incidence of SA-AKI. Secondary outcomes included ICU mortality, in-hospital mortality, 1-year all-cause mortality, mechanical ventilation rate, renal replacement therapy rate, and use of vasoactive drugs.

### Data extraction and preprocessing

The following variables were extracted from the database, including patient demographics, vital signs, medical history, laboratory tests, and scoring data. Organ support data included the use of vasoactive drugs, mechanical ventilation, and renal replacement therapy. Outcome variables included the occurrence of SA-AKI within 7 days after ICU admission, ICU length of stay, ICU mortality rate, hospital length of stay, hospital mortality rate, and 1-year all-cause mortality rate. Considering that some laboratory data may be measured multiple times within 24 h, this study extracted the first value of the day for those variables. For missing experimental data that accounted for less than 15% of the total population size, multiple imputation was used for handling [24, 25].

### Statistical methods

All analyses in this study were conducted in two cohorts: unmatched and propensity score-matched. Baseline patient characteristics were stratified by gender. Normally distributed continuous data are presented as mean ± standard deviation (X ± s), while non-normally distributed continuous data are presented as median (interquartile range) [Median (IQR)]. Group comparisons were performed using t-tests or rank-sum tests. Categorical data are presented as frequency (N) and percentage (%), with group comparisons analyzed using chi-square tests. Propensity scores were calculated for gender and matched 1:1, The variables for propensity matching include age, BMI, race, admission type, microorganisms, infection sources, comorbidities, interventions [33]. Multivariable Cox regression or logistic regression was used to assess the association between gender and the incidence of SA-AKI, organ support rate, ICU mortality rate, hospital mortality rate, and 1-year all-cause mortality rate. All analyses were performed using R software version 4.62.

## Result

### Baseline characteristics of the population

**Fig. 1:**
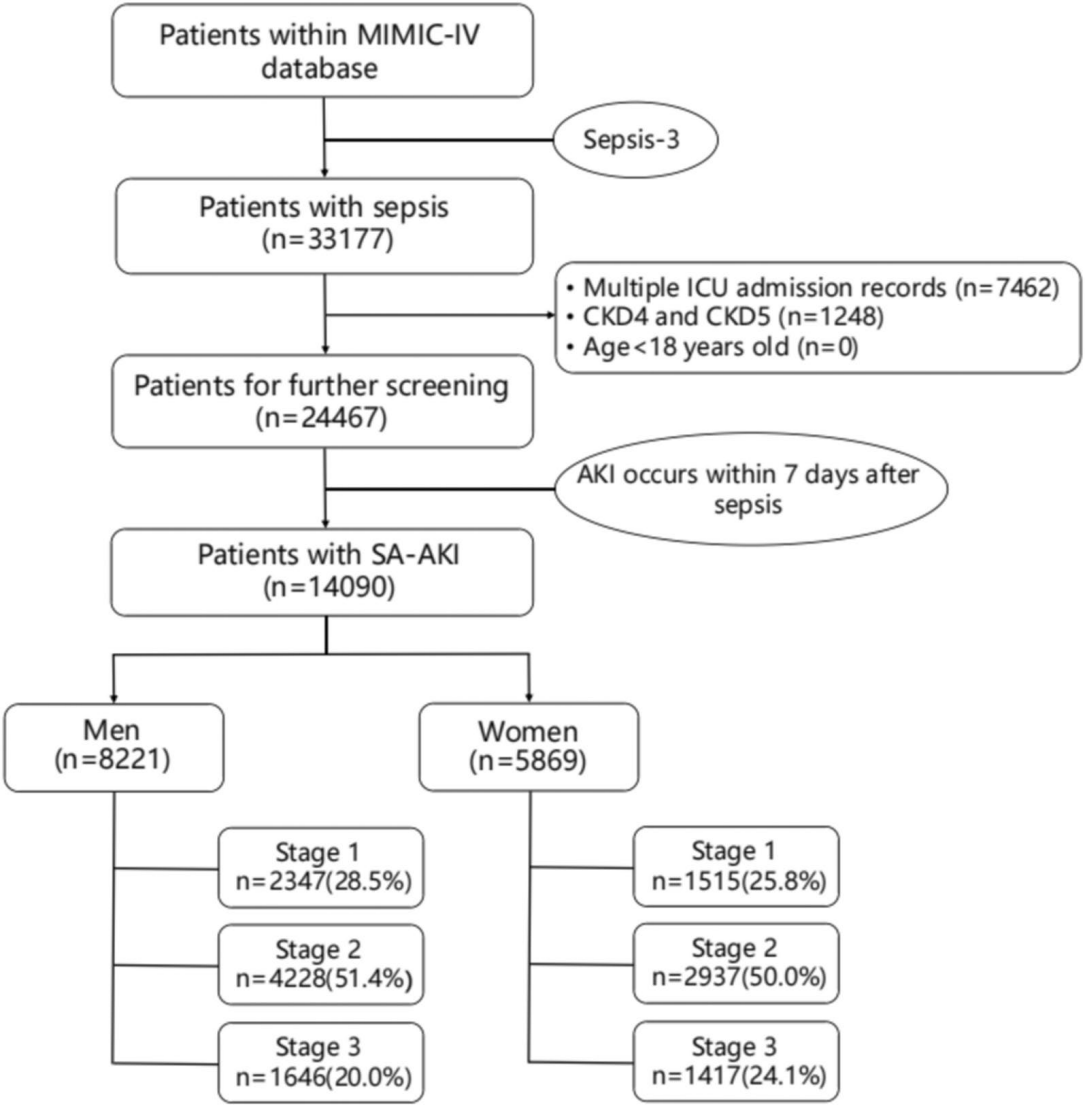
Study flow chart. *ICU* intensive care unit, *CKD* Chronic kidney disease, *SA-AKI* Sepsis associated acute kidney injury

As shown in Fig. 1, a total of 33,177 sepsis patients were included in this study. Patients with multiple repeated ICU admissions and those requiring kidney dialysis treatment for CKD stage 4 or above were excluded.24467 patients with sepsis were ultimately included in the analysis, and 14,090 patients developed AKI within 7 days after the onset of sepsis, including 8221 males and 5869 females. The baseline demographic and clinical biochemical characteristics are presented in Table 1. In the unmatched cohort, there was no significant difference between males and females in terms of BMI. Females had a higher age than males (70.2 [58.0–81.3] vs. 66.5 [55.6–77.1], *P* < 0.001). Male patients had a higher proportion of emergency department admissions compared to female patients (16.3% vs. 13.1%, *P* < 0.001). The prevalence rates of coronary heart disease (35.4% vs. 22.4%, *P* < 0.001), peripheral vascular disease (12.7% vs. 10.6%, *P* < 0.001), chronic kidney disease (19.5% vs. 16.6%, *P* < 0.001), chronic liver disease (11.1% vs. 8.23%, *P* < 0.001), and cancer (15.3% vs. 13.0%, *P* < 0.001) were higher among male patients. Female patients had a higher prevalence rate of hypertension (44.8% vs. 43.5%, *P* = 0.041), chronic heart failure (29.3% vs. 27.1%, *P* < 0.001), cardiovascular diseases (15.6% vs. 13.5%, *P* = 0.001), and chronic lung diseases (30.4% vs. 23.0%, *P* < 0.001). In terms of infection sources, female patients had a higher prevalence rate of pulmonary infections (42.9% vs. 39.5%, *P* < 0.001), gastrointestinal infections (16.4% vs. 13.6%, *P* < 0.001), and urinary tract infections (27.5% vs.13.3%, *P* < 0.001) compared to male patients. Male patients had a higher prevalence rate of catheter-related infections compared to female patients (8.48% vs. 7.14%, *P* < 0.001). In the matched cohort, female patients were older than male patients, while other baseline demographic characteristics were generally consistent (Table 1).

**Table 1:** Baseline characteristics of patients grouped by gender

N	Unmatched cohort			Matched cohort		
	Female (*N* = 10,380)	Male (*N* = 14,087)	*P*	Female (N = 9064)	Male (N = 9064)	*P*
Age (years)	70.2 [58.0;81.3]	66.5 [55.6;77.1]	< 0.001	68.8 [56.8;80.1]	68.0 [57.1;78.7]	0.010
BMI (kg/m^2^)	27.5 [23.2;33.2]	27.6 [24.2;31.8]	0.622	27.4 [23.2;33.0]	27.6 [24.0;31.8]	0.327
Ethnicity, *n*%			< 0.001			0.897
White	6963 (67.1%)	9581 (68.0%)		6100 (67.7%)	6067 (67.3%)	
Asian	256 (2.47%)	355 (2.52%)		227 (2.52%)	240 (2.66%)	
Black	1062 (10.2%)	934 (6.63%)		802 (8.90%)	799 (8.87%)	
Other	2099 (20.2%)	3217 (22.8%)		1883 (20.9%)	1906 (21.1%)	
Admission type, *n*%			< 0.001			0.389
Emergency	1359 (13.1%)	2298 (16.3%)		7740 (85.9%)	7726 (85.7%)	
Non-emergency	9017 (86.9%)	11,787 (83.7%)		1272 (14.1%)	1286 (14.3%)	
Infection sources, *n*%						
Lung	4449 (42.9%)	5558 (39.5%)	< 0.001	3842 (42.6%)	3789 (42.0%)	0.433
Intestinal	1698 (16.4%)	1922 (13.6%)	< 0.001	1415 (15.7%)	1422 (15.8%)	0.902
Urinary system	2855 (27.5%)	1880 (13.3%)	< 0.001	1880 (20.9%)	1755 (19.5%)	0.021
Catheter related	741 (7.14%)	1195 (8.48%)	< 0.001	668 (7.41%)	673 (7.47%)	0.902
Skin and soft tissue	527 (5.08%)	668 (4.74%)	0.241	456 (5.06%)	451 (5.00%)	0.892
Microorganisms, *n*%						
Gram-Negative	2825 (27.2%)	2998 (21.3%)	< 0.001	2196 (24.4%)	2166 (24.0%)	0.614
Gram-Positive	3279 (31.6%)	4322 (30.7%)	0.133	2800 (31.1%)	2799 (31.1%)	1.000
Fungi	2104 (20.3%)	2107 (15.0%)	< 0.001	1661 (18.4%)	1627 (18.1%)	0.524
Virus	124 (1.19%)	117 (0.83%)	0.005	0.00 [0.00;0.00]	0.00 [0.00;0.00]	0.431
Comorbidities, *n*%						
Hypertension	4655 (44.8%)	6131 (43.5%)	0.041	3974 (44.1%)	3938 (43.7%)	0.599
Coronary atherosclerosis	2329 (22.4%)	4990 (35.4%)	< 0.001	2254 (25.0%)	2262 (25.1%)	0.904
Chronic heart failure	3043 (29.3%)	3821 (27.1%)	< 0.001	2541 (28.2%)	2500 (27.7%)	0.507
Chronic liver disease	854 (8.23%)	1567 (11.1%)	< 0.001	805 (8.93%)	812 (9.01%)	0.876
Diabetes	2925 (28.2%)	4180 (29.7%)	0.011	2540 (28.2%)	2530 (28.1%)	0.881
Chronic kidney disease	1728 (16.6%)	2745 (19.5%)	< 0.001	1540 (17.1%)	1588 (17.6%)	0.355
Peripheral vascular disease	1102 (10.6%)	1795 (12.7%)	< 0.001	1010 (11.2%)	1066 (11.8%)	0.199
Cerebro vascular disease	1618 (15.6%)	1904 (13.5%)	< 0.001	1345 (14.9%)	1343 (14.9%)	0.983
Chronic pulmonary disease	3152 (30.4%)	3239 (23.0%)	< 0.001	2544 (28.2%)	2527 (28.0%)	0.791
Cancer	1345 (13.0%)	2150 (15.3%)	0.001	1248 (13.8%)	1289 (14.3%)	0.392
Severity scale						
APS III	50.0 [37.0;69.0]	48.0 [35.0;68.0]	< 0.001	50.0 [37.0;69.0]	49.0 [35.0;68.0]	0.007
SOFA	5.00 [3.00;8.00]	6.00 [4.00;9.00]	< 0.001	5.00 [3.00;8.00]	6.00 [4.00;8.00]	< 0.001
OASIS	35.0 [29.0;42.0]	34.0 [27.0;40.0]	< 0.001	35.0 [28.0;41.0]	34.0 [28.0;40.0]	< 0.001
SAPSII	38.0 [30.0;48.0]	37.0 [29.0;47.0]	< 0.001	38.0 [30.0;48.0]	38.0 [30.0;47.0]	0.140
Interventions						
KRT	784 (7.55%)	1148 (8.15%)	0.092	701 (7.78%)	679 (7.53%)	0.556
Mechanical ventilation	5693 (54.8%)	8666 (61.5%)	< 0.001	5105 (56.6%)	5118 (56.8%)	0.857
Vasopressor use	5028 (48.5%)	7443 (52.8%)	< 0.001	4457 (49.5%)	4472 (49.6%)	0.835
Diuretic	1776 (17.1%)	2435 (17.3%)	0.732	1555 (17.3%)	1472 (16.3%)	0.102
Statin	1137 (11.0%)	1795 (12.7%)	< 0.001	1020 (11.3%)	1011 (11.2%)	0.851
ACEI/ARBs	216 (2.08%)	307 (2.18%)	0.630	191 (2.12%)	190 (2.11%)	1.000
Contrast	248 (2.39%)	308 (2.19%)	0.313	210 (2.33%)	211 (2.34%)	1.000
Vancomycin	2342 (22.6%)	3262 (23.2%)	0.282	2083 (23.1%)	2017 (22.4%)	0.248
Aminoglycosides	150 (1.45%)	223 (1.58%)	0.414	133 (1.48%)	135 (1.50%)	0.951
Vital signs						
Heart rate(beats/min)	86.0 [76.0;98.0]	84.0 [75.0;96.0]	< 0.001	86.0 [76.0;98.0]	84.0 [74.0;97.0]	< 0.001
RR (times/min)	19.0 [16.0;22.0]	18.5 [16.0;22.0]	0.001	19.0 [16.0;22.0]	19.0 [16.0;22.0]	0.295
MAP (mmHg)	74.0 [68.0;81.0]	74.5 [69.0;81.5]	< 0.001	74.0 [68.0;81.0]	75.0 [69.0;82.0]	< 0.001
Spo2%	97.5 [96.0;99.0]	97.5 [96.0;99.0]	0.516	97.5 [96.0;99.0]	97.0 [96.0;99.0]	< 0.001
Temperature(℃)	36.8 [36.6;37.2]	36.9 [36.6;37.2]	< 0.001	36.8 [36.6;37.2]	36.9 [36.6;37.2]	< 0.001
Laboratory tests						
BUN (mg/dL)	19.0 [13.0;31.0]	20.0 [14.5;33.0]	< 0.001	19.0 [12.5;30.0]	21.0 [15.0;34.0]	< 0.001
Creatinine(mg/dL)	0.90 [0.65;1.35]	1.10 [0.80;1.60]	< 0.001	0.90 [0.65;1.35]	1.10 [0.80;1.60]	< 0.001
Sodium(mEg/L)	139 [136;141]	138 [136;141]	< 0.001	138 [136;141]	138 [136;141]	0.353
Potassium(mEq/L)	4.05 [3.75;4.45]	4.20 [3.90;4.60]	< 0.001	4.10 [3.75;4.45]	4.20 [3.90;4.60]	< 0.001
Chloride(mEq/L)	105 [101;108]	105 [101;108]	0.695	105 [101;108]	105 [101;108]	0.010
PT(s)	14.0 [12.4;16.4]	14.2 [12.8;16.4]	< 0.001	13.9 [12.4;16.3]	14.2 [12.8;16.4]	< 0.001
APTT(s)	30.9 [27.0;38.8]	31.5 [27.7;38.5]	< 0.001	30.6 [26.9;37.6]	31.0 [27.4;37.0]	0.003
Platelets(k/UL)	194 [134;266]	171 [124;232]	< 0.001	192 [132;265]	175 [126;238]	< 0.001
WBC(k/UL)	11.7 [8.40;15.9]	11.8 [8.50;15.8]	0.843	11.8 [8.40;15.8]	11.8 [8.45;15.8]	0.956
Hemoglobin(g/UL)	10.2 [9.00;11.5]	10.8 [9.40;12.3]	< 0.001	10.2 [9.00;11.5]	10.9 [9.40;12.4]	< 0.001
Lactate(mmol/L)	1.70 [1.20;2.60]	1.80 [1.30;2.60]	0.002	1.75 [1.20;2.60]	1.75 [1.25;2.55]	0.656
Proteinuria(mg/dL)	30.0 [30.0;100]	30.0 [30.0;100]	0.472	30.0 [30.0;100]	30.0 [30.0;100]	0.117
NLR	8.89 [4.94;15.8]	8.49 [4.82;15.4]	0.034	8.82 [4.87;15.7]	8.92 [5.02;16.2]	0.043
Pao2/Fio2	228 [163;314]	229 [164;311]	0.875	228 [162;314]	226 [161;308]	0.060
PO2(mmHg)	118 [85.0;184]	126 [89.0;198]	< 0.001	120 [85.9;187]	120 [86.0;186]	0.776
BE (mmol/L)	0.00 [-3.50;1.00]	0.00 [-3.00;1.00]	0.189	-0.50 [-3.50;1.00]	0.00 [-3.00;1.00]	0.004
Bicarbonate(mmol/L)	22.5 [20.0;25.0]	23.0 [20.5;25.0]	< 0.001	22.5 [20.0;25.0]	23.0 [20.5;25.0]	< 0.001
SA-AKI	5869 (56.5%)	8221 (58.4%)	0.005	5063 (56.2%)	5207 (57.8%)	0.031
AKI stage			< 0.001			< 0.001
KDIGO 1	1810 (17.4%)	2825 (20.1%)		1597 (17.7%)	1704 (18.9%)	
KDIGO 2	3660 (35.3%)	5198 (36.9%)		3159 (35.1%)	3371 (37.4%)	
KDIGO 3	2106 (20.3%)	2519 (17.9%)		1776 (19.7%)	1647 (18.3%)	

*BMI* Body mass index, *ACEI/ARBs* Angiotensin-converting enzyme inhibitors/angiotensin receptor blockers, *APS III* Acute Physiology Score III, *OASIS* Oxford Acute Severity of Illness Score, *SOFA* Sequential Organ Failure Assessment, *SAPS* Simplified acute physiology score, *SIRS* Systemic inflammatory response syndrome, *KRT* Kidney replacement therapy, *RR* Respiratory rate, *MAP* mean arterial pressure, *APTT* Activated partial thromboplastin time, *PT* Prothrombin time, *BUN* Blood urea nitrogen, *WBC* White blood cell, *SA-AKI* Sepsis Associated Acute kidney injury

### Association of sex with risk of sepsis associated acute kidney injury

**Fig. 2:**
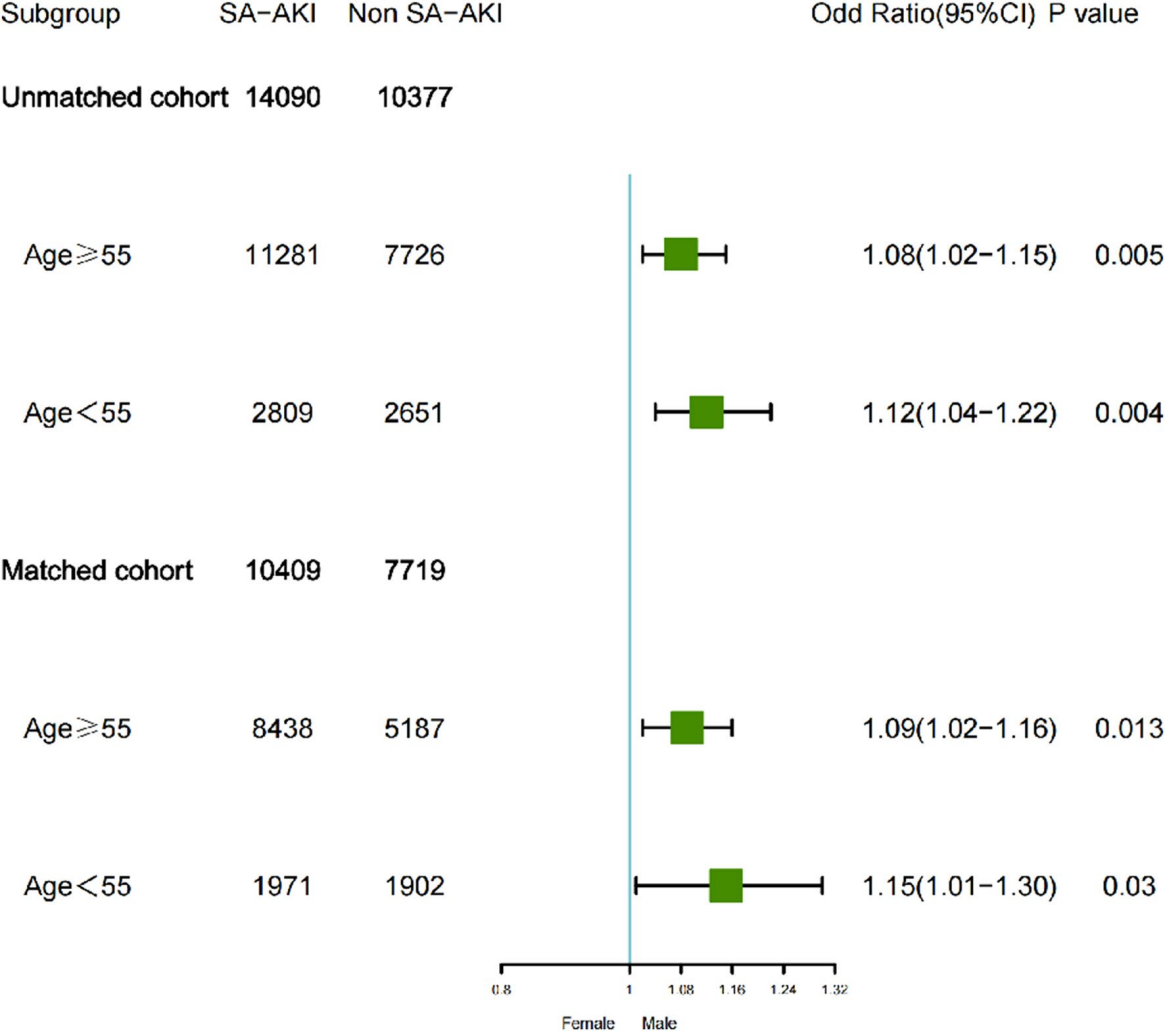
Association between sex and SA-AKI in age-stratified matched and unmatched cohorts. *SA-AKI* sepsis-related acute kidney injury

In the unmatched cohort, a total of 14,090 cases (57.1%) of sepsis-associated acute kidney injury (SA-AKI) occurred in ICU patients. The incidence rate of SA-AKI was higher in males than females (58.4% vs. 56.5%, *P* = 0.005), with the majority of patients in both groups classified as AKI stage 1–2 (males: 79.9%, females: 75.8%). In Table 2, we conducted logistic regression analysis on the occurrence of SA-AKI, and found that males had a higher risk associated with SA-AKI in the unmatched cohort (OR: 1.08; 95% CI 1.02–1.13; *P* < 0.001) (Model 1). After adjusting for age, BMI, race, infection source, admission type, comorbidities, treatment and laboratory variables, the association between males and SA-AKI remained significant (OR: 1.07; 95% CI 1.01–1.13; *P* = 0 0.028) (Model6). This association was also observed in propensity-matched cohort analysis indicating that male patients still had a higher risk of developing SA-AKI (Table 2). Subgroup analyses were further performed separately in matched and unmatched cohorts by stratifying age into ≥ 55 years old and < 55 years old categories which showed that male patients had a higher incidence rate of SA-AKI compared to female patients across different age strata (Fig. 2).

**Table 2:** Logistic regression modelled analysis for the association of male sex with sepsis associated acute kidney injury

	Unmatched cohort		Matched cohort	
	OR (95% CI)	*P*	OR (95% CI)	*P*
Model 1	1.08 (1.02–1.13)	< 0.001	1.07 (1.01–1.14)	0.026
Model 2	1.11 (1.06–1.17)	< 0.001	1.07 (1.01–1.14)	0.024
Model 3	1.11 (1.06–1.17)	< 0.001	1.08 (1.01–1.14)	0.017
Model 4	1.10 (1.05–1.17)	< 0.001	1.08 (1.01–1.14)	0.016
Model 5	1.08 (1.02–1.14)	< 0.001	1.08 (1.01–1.14)	0.017
Model 6	1.07 (1.01–1.13)	0.028	1.07 (1.01–1.14)	0.033

*SA-AKI* Sepsis associated acute kidney injury, *BMI* Body mass index, *CI* confidence intervals, *OR* odds ratio

Model 1, unadjusted; Model 2, adjusted for age; Model 3, adjusted for adjusted for age, BMI, race, admission type, infection resources, microorganisms; Model 4, adjusted for age, race, BMI, admission type, infection resources, microorganisms, hypertension, coronary atherosclerosis, chronic heart failure, chronic liver disease, diabetes,chronic kidney disease, peripheral vascular disease, chronic pulmonary disease, cancer; Model 5, adjusted for age, race, BMI, admission type, infection resources, microorganisms, hypertension, coronary atherosclerosis, chronic heart failure, chronic liver disease, diabetes, chronic kidney disease, peripheral vascular disease, chronic pulmonary disease, cancer, KRT, Mechnical ventilation, Vasopressor use, Diuretic, Statin, ACEI/ARBs, Contrast, Vancomycin, Aminoglycosides; Model 6, adjusted for age, race, BMI, admission type, infection resources, microorganisms, hypertension, coronary atherosclerosis, chronic heart failure, chronic liver disease, diabetes, chronic kidney disease, peripheral vascular disease, chronic pulmonary disease, cancer, KRT, Mechnical ventilation, Vasopressor use, Diuretic, Statin, ACEI/ARBs, Contrast, Vancomycin, Aminoglycosides, apsiii, sofa, oasis, sapsII, BUN, Creatinine, Potassium, Chloride, PT, Hemoglobin, Lactate, NLR, Proteinuria, Pao2/Fio2, PO2, Bicarbonate, MAP, Temperature

### Association of sex with mortality after sepsis associated acute kidney injury

**Fig. 3:**
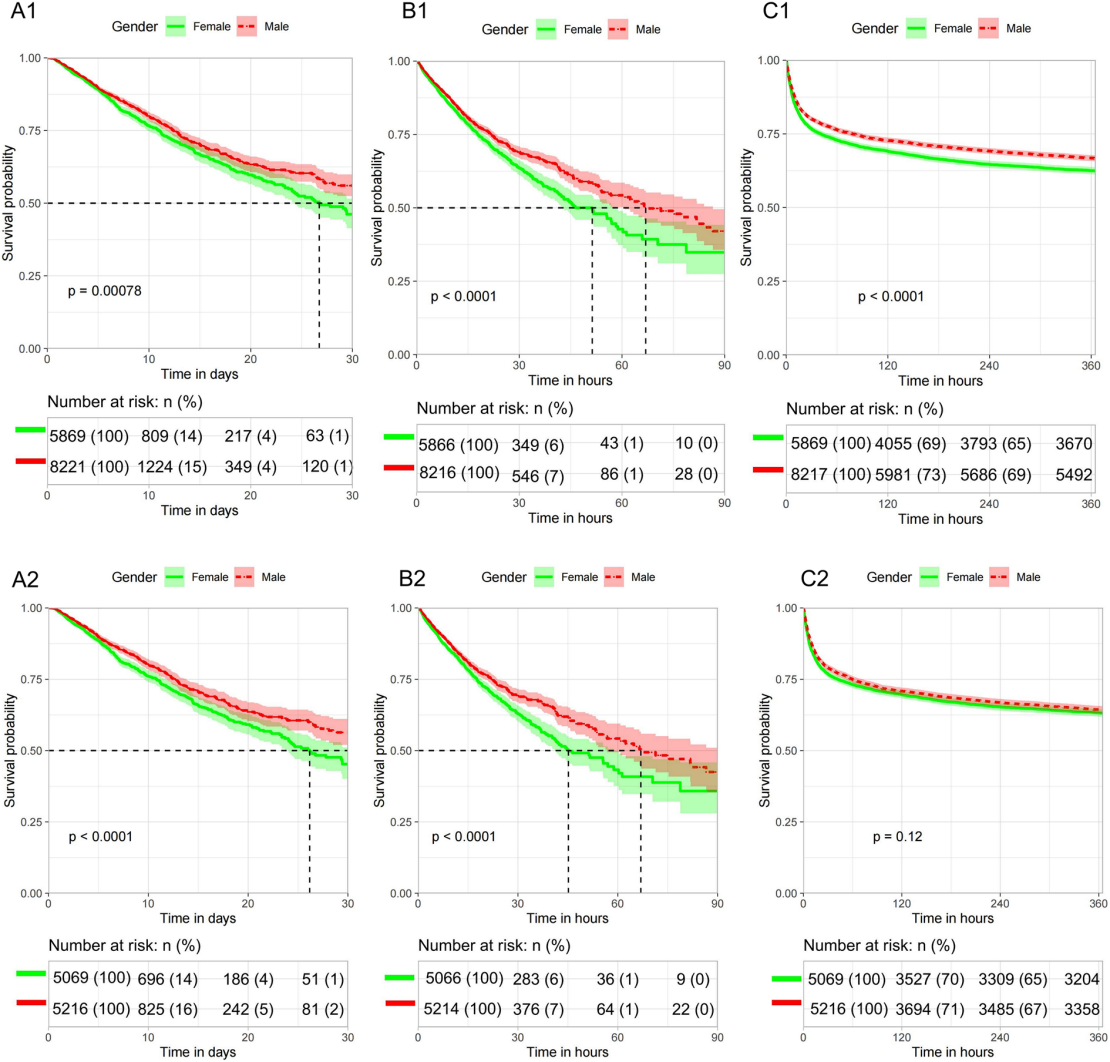
Kaplan–Meier curves in male and female patients with SA-AKI during different periods. **A1**–**C1**: 30 days in ICU, 90 days in hospital, 1-year follow-up periods in unmatched cohort. **A2**–**C2**: 30 days in ICU, 90 days in hospital, 1-year follow-up periods in matched cohort

In the unmatched SA-AKI patient cohort, the ICU mortality (13.5% vs. 11.6%, *P* = 0.001), in-hospital mortality (19.0% vs. 16.3%, *P* < 0.001), and 1-year all-cause mortality (37.6% vs. 33.3%, P < 0.001) were higher in female patients compared to male patients (Table 3). Kaplan–Meier analysis showed that male patients had significantly higher 30-day ICU survival rate (log rank *P* < 0.001), 90-day hospital survival rate (log rank *P* < 0.001), and 1-year survival rate (log rank P < 0.001) than female patients (Fig. 3). Cox regression analysis demonstrated that male patients had a lower risk of ICU death, hospital death, and 1-year all-cause death compared to female patients, with hazard ratios of 0.851[95% CI (0.774–0.935), *P* < 0.001], 0.845[95% CI (0.780–0.915)], *P* < 0.001], and 0.858[95% CI (0.811–0.907)], *P* < 0.001] respectively. After adjusting for age, BMI, race, infection source, admission type, comorbidities, interventions, severity scale and laboratory variables, male patients had a lower risk of ICU mortality, in-hospital mortality, and all-cause mortality within 1 year, the hazard ratios were 0.861[95% CI (0.778–0.952), *P* = 0.003], 0.875[95%CI (0.803–0.953), *P* = 0.002], and 0.985 [95% CI (0.827–1.05)], *P* = 0.61] respectively (Table 4). In the matched cohort population, male patients still had independent associations with reduced risks of ICU death and hospital death. However, the 1-year all-cause mortality in female patients, although higher than that in male patients, was not statistically significant (36.9% vs. 35.8%, *P* = 0.12) (Tables 3, 4, Fig. 3).

**Table 3:** Comparisons of ICU mortality, In-hospital mortality and 1-year all-cause mortality in male and female patients with SA-AKI

N	Unmatched cohort		*P*	Matched cohort		*P*
	Female	Male		Female	Male	
	5869	8221		5129	5129	
ICU mortality in 30 days (*n*, %)	790 (13.5%)	957 (11.6%)	< 0.001	703 (13.9%)	622 (11.9%)	< 0.001
In-hospital mortality in 90 days (*n*, %)	1115 (19.0%)	1342 (16.3%)	< 0.001	970 (19.1%)	877 (16.8%)	< 0.001
All-cause mortality in 1 year (*n*, %)	2204 (37.6%)	2739 (33.3%)	< 0.001	1870 (36.9%)	1867 (35.8%)	0.12

*SA-AKI* sepsis associated acute kidney injury, *ICU* Intensive Care Unit

**Table 4:** Crude and adjusted Hazard rate for mortality for men compared to women

		ICU mortality	*P*	In-hospital mortality	*P*	One year mortality	*P*
		HR (95% CI)		HR (95% CI)		HR (95% CI)	
Unmatched cohort	Crude model	0.851 (0.774–0.935)	< 0.001	0.845 (0.780–0.915)	< 0.001	0.858 (0.811–0.907)	< 0.001
	Adjust model 1	0.890 (0.810–0.979)	0.016	0.887 (0.819–0.961)	0.003	0.922 (0.871–0.975)	0.004
	Adjust model 2	0.834 (0.757–0.919)	< 0.001	0.834 (0.768–0.905)	< 0.001	0.922 (0.868–0.976)	0.005
	Adjust model 3	0.861 (0.778–0.952)	0.003	0.875 (0.803–0.953)	0.002	0.985 (0.927–1.05)	0.61
Matched cohort	Crude model	0.803 (0.721–0.894)	< 0.001	0.820 (0.748–0.899)	< 0.001	0.951 (0.891–1.01	0.121
	Adjust model 1	0.799 (0.717–0.891)	< 0.001	0.818 (0.747–0.897)	< 0.001	0.962 (0.902–1.03)	0.240
	Adjust model 2	0.805 (0.722–0.897)	< 0.001	0.810 (0.739–0.888)	< 0.001	0.930 (0.872–0.992)	0.027
	Adjust model 3	0.836 (0.746–0.937)	0.002	0.853 (0.775–0.938)	0.003	0.986 (0923–1.05)	0.681

Crude model unadjusted

Model 1 adjusted age

Model 2 adjusted age, BMI, race, Infection sources, comorbidity

Model 3 adjusted age, BMI, race, Infection sources, comorbidity, Severity scale, KRT, mechanical ventilation, Vasopressor use, ACEI/ARBs, Aminoglycosides, Diuretic, vancomycin, Vancomycin, BUN, Creatinine, Potassium, Chloride, PT, Hemoglobin, Lactate, NLR, Proteinuria, Pao2/Fio2, PO2, Bicarbonate, MAP, Temperature

*BMI* body mass index, *CI* confidence intervals, *HR* Hazard ratio

### Association of sex with organ supports after sepsis associated acute kidney injury

**Fig. 4:**
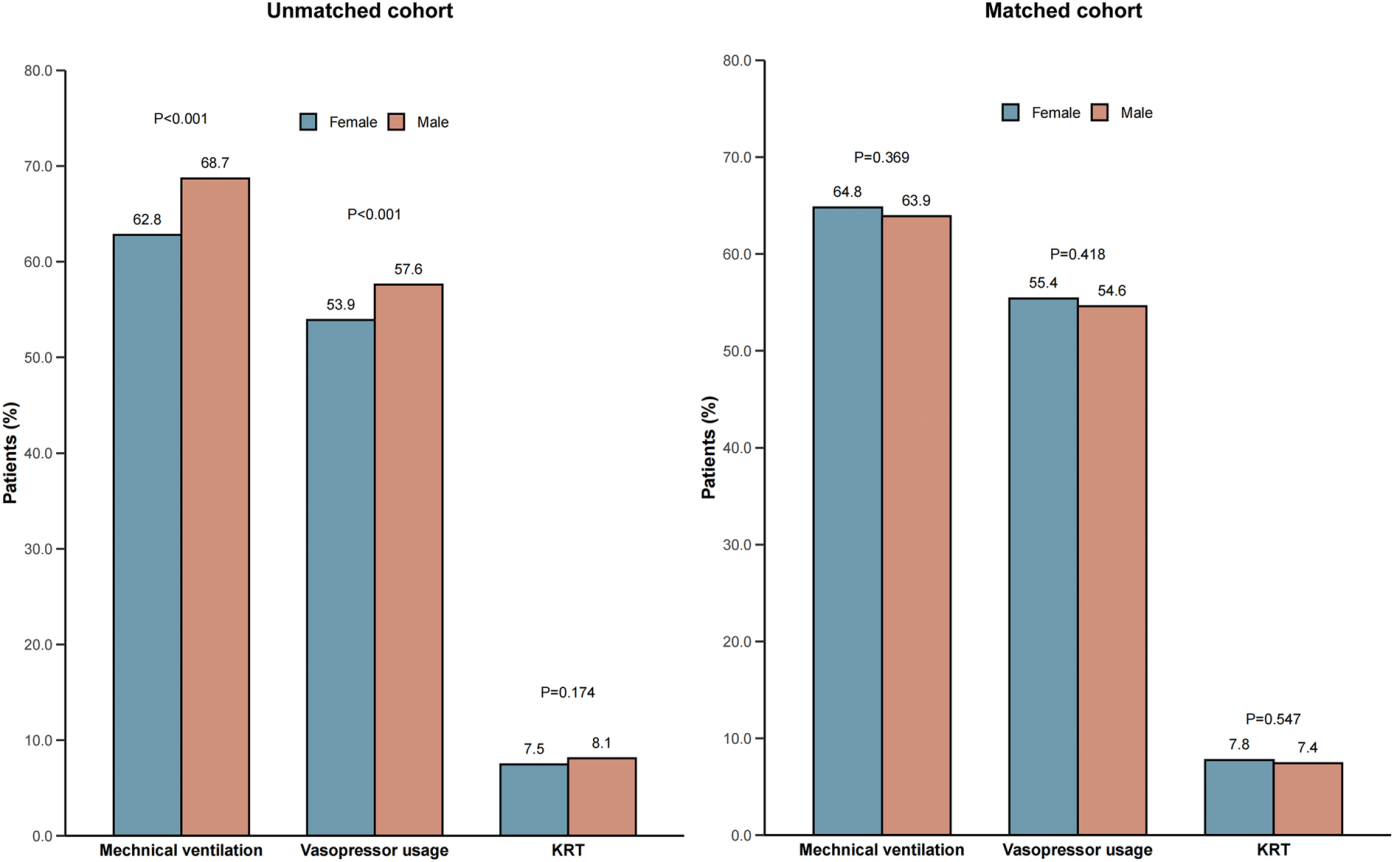
Comparison of organ support rates between males and females in SA-AKI patients. *KRT* Kidney replacement therapy

Figure 4 shows a comparison of organ support rates between male and female patients with SA-AKI. In the unmatched cohort, the rate of mechanical ventilation support and use of vasoactive drugs was higher in male patients than in female patients (*P* < 0.001). However, there was no statistically significant difference in the rate of kidney replacement therapy support between male and female patients, although it remained higher in males (*P* = 0.174) even after adjusting for variables. After adjustment, male patients still had higher rates of mechanical ventilation support (OR, 1.15; 95% CI 1.04–1.29; *P* < 0.001) and vasoactive drug use (OR, 1.10; 95% CI 1.01–1.20; *P* = 0.03) compared to female patients (Table 5). However, in the matched cohort, there were no statistically significant differences between male and female patients in terms of kidney replacement therapy, mechanical ventilation treatment or vasoactive drug use (Fig. 4).

**Table 5:** Unadjusted and adjusted odds ratio for organ support for men compared to women

		KRT		MV		VD	
		OR (95% CI)	*P*	OR (95% CI)	*P*	OR (95% CI)	*P*
Unmatched cohort	Unadjusted	1.09 (0.96–1.24)	0.16	1.30 (1.22–1.40)	< 0.001	1.16 (1.08–1.24)	0.006
	Adjusted	–	–	1.15 (1.04–1.29)	< 0.001	1.10 (1.01–1.20)	0.03
Matched cohort	Unadjusted	0.95 (0.82–1.10)	0.52	0.96 (0.89–1.04)	0.35	0.97 (0.89–1.05)	0.40
	Adjusted	–	–	0.95 (0.86–1.04)	0.27	1.10 (0.99–1.22)	0.53

*SA-AKI* sepsis associated acute kidney injury, *KRT* Kidney Replacement Therapy, *MV* Mechanical Ventilation, *VD* Vasopressor Drug

Adjusted variations: age, race, BMI, admission type, infection resources, microorganisms, hypertension, coronary atherosclerosis, chronic heart failure, chronic liver disease, diabetes, chronic kidney disease, peripheral vascular disease,chronic pulmonary disease, cancer, KRT, Mechnical ventilation, Vasopressor use, Diuretic, Statin, ACEI/ARBs, Contrast, Vancomycin, Aminoglycosides, apsiii, sofa, oasis, sapsII, BUN, Creatinine, Potassium, Chloride, PT, Hemoglobin, Lactate, NLR, Proteinuria, Pao2/Fio2, PO2, Bicarbonate, MAP, Temperature

## Discussion

In this propensity-matched cohort study based on the MIMIC-IV database, the incidence of female SA-AKI during ICU hospitalization was lower than that of males. Females were associated with higher ICU and in-hospital mortality, but there was no statistically significant difference in 1-year all-cause mortality between the two groups. Among SA-AKI patients, there were no significant differences between males and females in terms of renal replacement therapy rate, mechanical ventilation support rate, and vasopressor use rate.

In our study, univariate analysis revealed that the incidence of SA-AKI in female patients with sepsis in the ICU was lower than that in male patients. Even after adjusting for age, BMI, race, infection source, admission type, comorbidities, treatment and laboratory variables, we still found that female patients with sepsis had a lower risk of developing SA-AKI compared to males. Furthermore, even after propensity score matching analysis on the population, we obtained the same results. However, our study design does not allow us to provide detailed explanations for the possible pathophysiological reasons behind this finding. SA-AKI is a complex clinical syndrome with intricate mechanisms of onset. Immune dysfunction combined with high levels of circulating endotoxins and cytokines accelerates the progression of SA-AKI [26]. Previous studies have also found gender differences in the incidence of sepsis. Adrie et al. discovered a lower incidence rate of severe sepsis among females [27]. Wichmann et al. found a significantly lower occurrence rate of severe sepsis/septic shock in female ICU patients aged 60–79 compared to male patients [28]. Additionally, Sperry et al. also observed a significant decrease in multiple organ failure and hospital infection rates among females [29]. This may be attributed to hormonal levels and other characteristics affecting immune function and inflammation levels in female patients; elevated estrogen levels may enhance immune function [30–32], while an advantage in anti-inflammatory mediators provides protection against critical sepsis for females [30]. These factors may contribute to reducing susceptibility to SA-AKI among females.

There are many factors in clinical practice that are independently associated with poor prognosis of SA-AKI [33–36]. In this article, we also reported the gender differences in the outcomes of SA-AKI. In both unmatched and matched cohorts, we found that females had an increased independent risk of ICU mortality and in-hospital mortality. There have been fewer reports on gender-related outcomes in SA-AKI, but there has been sufficient attention given to the differences in outcomes based on gender in sepsis, although conflicting results have been obtained. Schröder et al. found that female patients with surgical sepsis had a higher survival rate than males [37]. However, this study was limited to surgical sepsis patients and included a small number of participants. Adrie et al., in a large case–control study including 1692 critically ill septic patients, also reported similar results [38]. Some basic experimental studies have also found that female animals with sepsis have better survival rates compared to males [39]. The increase in pro-inflammatory cytokine levels is believed to be the cause of this phenomenon, and sex steroids can regulate inflammatory responses and may subsequently affect post-sepsis outcomes [40].

Our research results are contrary to some previous studies [30, 37]. We found that women have a higher risk of ICU mortality and in-hospital mortality independently associated with SA-AKI patients. Our findings are consistent with those of Shapati et al., who demonstrated that women are an independent predictor for increased mortality in critically ill patients with infection [41]. Recently, Combes et al. analyzed gender-related outcomes in a mixed population of patients with hospital-acquired infections in the ICU and reported an increased risk of ICU mortality among women [42]. Previous studies have reported differences in care between male and female patients during hospitalization, with male patients potentially receiving better care [43]. In addition, our study found that female patients had higher severity scores upon admission to the ICU. Female APS III (50.0 [37.0–69.0] vs. 48.0[35.0–68.0], *P* < 0.001), SAPS II (38.0 [30.0–48.0] vs. 37.0 [29.0–47.0], *P* < 0001), OASIS (35.0[29.0–42.0] vs. 34.0 [27.0–40.0], *P* < 0.001) were all higher than males, which may contribute to the increased mortality rate among females during their stay in the ICU as well as throughout their hospitalization period.

In this study, we explored the issue of gender differences in organ support among SA-AKI patients. In the unmatched cohort, female patients had a lower likelihood of receiving mechanical ventilation and vasopressor therapy. However, in the matched cohort, there was no statistically significant difference between males and females. Furthermore, there were no gender-related statistical differences in kidney replacement therapy observed in both matched and unmatched cohorts. Previous studies have found gender differences in organ support among critically ill patients in the ICU [44, 45]. A recent meta-analysis showed that female patients had a lower proportion of receiving organ support (including kidney replacement therapy and mechanical ventilation) during ICU treatment compared to males [46]. However, this study had substantial heterogeneity and potential bias within its sample population. In contrast, our study focused specifically on SA-AKI patients and concluded that there were no gender differences associated with organ support within this specific population. Additionally, our findings are consistent with another meta-analysis regarding organ support during hospitalization for sepsis patients [47]. However, it is worth noting that we did not obtain information such as pre-admission care, discussions or changes during ICU period or decision-makers for substitution therapies nor other social factors that may influence decisions regarding provision of organ support within our cohort. Therefore, further research is needed to better understand the impact of gender differences on rates of organ support in the ICU setting.

Our study has many advantages. It is an observational study based on a large database, using propensity score matching and non-matching cohort analysis to evaluate gender differences in the incidence of SA-AKI, ICU mortality, in-hospital mortality, and organ support rate among the included population. Propensity scores matching analysis retained enough patients and significantly reduced bias and standardized differences in important demographic and clinical characteristics between male and female patients. Additionally, we used the latest definition of SA-AKI [5] to comprehensively describe for the first time the gender differences in SA-AKI in the ICU setting. However, our study also has some limitations. Firstly, it is an observational retrospective design based on a database that only considers traditional parameters without including certain gender-specific variables such as hormone levels which may help explain potential mechanisms underlying our findings. Secondly, decisions regarding initiation and withdrawal of life-sustaining treatments in the ICU are multifactorial. While we attempted to balance clinical factors that could influence this decision-making process, we lack information on social factors such as gender roles or socioeconomic status that could confound this relationship. Although we were able to significantly reduce standardized differences for many important confounding factors by using propensity score matching, there still remains some variability between male and female patients that cannot be balanced within our model and must be excluded from propensity scores. Therefore, there may be residual confounding factors beyond our control present in our analysis. Furthermore, when preadmission creatinine was missing, the first creatinine on admission was used. As we did not use the lowest creatinine value during the entire ICU stay, this may have led to an underestimation of AKI, especially in emergency cases. Lastly, our study is limited to septic patients in ICUs; therefore, caution should be exercised when attempting to generalize our findings to the entire population.

## Conclusions

In the ICU setting, male patients have a higher incidence of SA-AKI, while female SA-AKI patients face a higher risk of ICU mortality and in-hospital mortality. Gender may affect the incidence rate and clinical outcomes of SA-AKI, further research is needed to replicate our research and fully understand the impact of gender on SA-AKI patients.

## Data Availability

Data are available upon reasonable request.
